# Comparison of Cost-Effectiveness and Cost-Utility of a Telerehabilitation Programme for the Management of Non-Specific Neck Pain: A Study Protocol

**DOI:** 10.3390/healthcare13233076

**Published:** 2025-11-26

**Authors:** Cristian Sánchez-Ferre, Adelaida María Castro-Sánchez, Inmaculada Carmen Lara Palomo, Fernando Reche-Lorite, Rosario de la Torre-Olivares, Manuel Fernández-Sánchez, Manuel Saavedra-Hernández

**Affiliations:** 1 Department of Nursing, Physical Therapy and Medicine, University of Almeria, 04120 Almeria, Spain; csf088@inlumine.ual.es (C.S.-F.); ilp813@ual.es (I.C.L.P.); freche@ual.es (F.R.-L.); manuelf@ual.es (M.F.-S.); saavedra@ual.es (M.S.-H.); 2 Westen Health District, Servicio Andaluz de Salud, El Ejido, 04005 Almeria, Spain; rosario.torre.sspa@juntadeandalucia.es

**Keywords:** neck pain, telemedicine, exercise, cost-effectiveness, cost-utility

## Abstract

**Background/Objectives**: Neck pain is a complex biopsychosocial condition that affects a significant proportion of the global population. Projections indicate that over the next 30 years, cervical pain will impact approximately 269 million individuals, positioning it as one of the primary drivers of healthcare expenditure and system burden worldwide. While exercise is a key approach for neck pain, the effectiveness and cost-effectiveness of treatments delivered remotely remain underexplored. The aim of this study is to analyse the cost-effectiveness and cost-utility of the treatment of non-specific neck pain through a telerehabilitation programme based on cervical exercise and analgesic electrotherapy. **Methods**: This is a study protocol for an economic evaluation based on a randomised controlled trial (RCT) with a sample size of 210 participants (*N* = 210). The sample will be evenly divided into two groups to perform the same cervical exercise programme combined with electroanalgesia: one group will receive the intervention via telerehabilitation, and the other will receive the intervention face-to-face. Three sessions will be held per week for eight weeks for a total of 24 sessions. Demographic and clinical data of the participants, healthcare resource utilisation, and associated costs will be collected. Assessments will be carried out throughout the study: before the first session (baseline), at 8 weeks (post-intervention), and 6 months after completion of the treatment (follow-up). **Discussion**: This study will make a significant contribution to reducing costs and improving the treatment of patients with non-specific chronic neck pain. The social perspective associated with economic evaluation will enable the investigation of indirect costs such as work absenteeism or expenses borne by the patient, providing useful data to optimise planning and decision-making in the healthcare sector.

## 1. Introduction

Neck pain (NP) is a complex biopsychosocial condition associated with a reduction in quality of life, decreased work productivity, limitation of activities of daily living, and increased use of healthcare services [1]. It is estimated to affect between 20 and 70% of the adult population over the course of their lives [2]. The prevalence of NP is indeed high worldwide, mainly affecting the working-age population (peaking between 45 and 74 years) and impacting women to a greater extent both in terms of prevalence and years lived with disability [3,4]. While acute NP usually resolves within two months, approximately 50% of individuals report persistent pain after one year [5], and up to 60% of the working population experience a recurrence in the year following their initial episode [6]. If the duration of symptoms persists for 12 weeks or more [7], it acquires the status of chronic pain, and if pathognomonic signs and symptoms have also been ruled out, it may be classified as non-specific pain [8]. The combination of both gives rise to non-specific chronic neck pain (NSCNP), a common condition that has a significant socioeconomic impact [8]. The Global Burden of Disease 2021 analysis ranked NP 11th out of 369 causes of years lived with disability, affecting 203 million people worldwide in 2020, including 27.66 million in Europe [4]. By 2050, the global prevalence is expected to rise by 32.5%, potentially affecting 109 million (CI: 88.8–131) men and 160 million (CI: 131–192) women [4].

NP is linked to impaired work performance and imposes a considerable economic burden on both individuals and healthcare systems. In the United States, it is estimated that there are approximately 10.2 million annual visits to physiotherapists or hospitals due to NP [1]. In Belgium, Gorasso et al. estimated the direct annual cost per patient at EUR 2212—36% higher than for individuals without NP [9]. Meanwhile, the Netherlands reported total costs of USD 686 million for related conditions [10]. Economic evaluations in healthcare distinguish between direct costs (related to the use of services and resources) and indirect costs (related to lost productivity, quality of life, and reduced independence). Indirect costs often exceed direct medical and pharmaceutical expenses [11]. The majority of health-related expenditure is caused by so-called presenteeism, which refers to reduced productivity due to attending work while ill and performing below usual standards, and is simultaneously the greatest risk factor for experiencing periods of sickness absence [12]. Economic losses resulting from presenteeism due to NP constitute a global problem affecting the world’s leading economies, with annual per capita losses of USD 1290 in the USA (an approximate total of USD 11.87 billion) and USD 414.05 per capita in Japan (an approximate total of USD 28 billion) [12,13]. A key factor in reducing the costs of NP treatment appears to be early intervention, as a delayed start to treatment may result in an increase in total costs of up to 117% [14]. To truly understand the economic impact of a disease, two main types of analysis can be used: cost-effectiveness and cost-utility. In the field of health economic evaluation, cost-effectiveness analysis allows decision-making and comparison of interventions by considering both the costs of a treatment and how effective that treatment is for at least two competing strategies [15], while cost-utility analysis also incorporates quality of life [16].

There is a consensus among the highest-quality European clinical guidelines regarding the recommendation of therapeutic exercise as an essential component in the treatment of chronic neck pain (CNP), both as a standalone intervention and as part of multimodal programmes alongside education, manual therapy, or psychological approaches [17]. Recent studies have identified exercise-based therapy as a cost-effective intervention for CNP [3]. Moreover, exercise has been shown to produce immediate analgesic effects, reflected in an increased pain threshold and modulation of nociceptive sensitivity [18]. In individuals with NSCNP, both isometric and joint mobility exercises have demonstrated hypoalgesic effects—not only at the site of pain but also in distant body regions [19]. The McKenzie method is a widely used strategy in the management of NP that has demonstrated its effectiveness in adult patients with moderate to severe pain [20]. It is based on a comprehensive assessment that integrates medical history, physical examination, and the patient’s response to repeated movements [21]. It also addresses cognitive–sensory dimensions of pain, such as fear-avoidance and negative expectations, and promotes self-management through the performance of specific, repetitive movements that reach maximum joint range, correcting dysfunctional motor patterns and reducing functional limitations [20,21,22,23].

Since its development in 1967 [24], transcutaneous electrical nerve stimulation (TENS) has remained one of the most widely adopted choices for the treatment of musculoskeletal pain [25,26]. TENS devices are portable and affordable, allowing low-intensity pulsed electrical currents to be applied via electrodes placed directly on the skin, stimulating peripheral nerves to produce a hypoalgesic effect [27]. The frequency, intensity, and pulse duration parameters can be individually adjusted to suit clinical needs [27]. Evidence suggests supporting the use of TENS (60–100 Hz) in NP [28,29]; especially when used in conjunction with other therapies, TENS may enhance tolerance to cervical exercises, facilitate mobility, and improve stretching capacity [30].

Self-management is a critical component in the treatment of chronic musculoskeletal pain, understood as the patient’s ability to actively manage their condition on a daily basis throughout the course of the condition [31]. This process involves interrelated tasks such as the medical management of pain, adaptation to affected social roles, and emotional grieving, all mediated by psychological functions that enable the individual to monitor their behaviour, set motivating short-term goals, and adapt to their context [31]. Current evidence suggests that musculoskeletal conditions such as CNP can be effectively managed in primary care through patient-centred approaches [32]. Recent clinical practice guidelines recommend, as a first-line therapeutic approach, the combination of education, exercise, and individualised counselling, with the aim of improving understanding of the condition, promoting autonomy in self-care, and reducing dependence on the healthcare system [32,33]. It is necessary to tailor the information to the characteristics and needs of each patient, addressing, in addition to the prognosis and clinical aspects, the psychosocial and functional variables that support active and sustained recovery [33]. Recently, innovative and promising treatment alternatives have emerged through new technologies, mobile devices, and telecommunications, enabling patients to participate more actively in their treatment, promoting self-care, correlating with improvements in pain, and making treatments via E-health a promising option in the management of chronic pain [34]. Telerehabilitation is a form of telemedicine that enables the assessment, treatment, and follow-up of rehabilitation programmes at a distance for patients with various conditions, with the added potential to reduce the costs associated with healthcare provision [35]. Although still a developing field, telerehabilitation for NSCNP has shown comparable—and in some cases superior—effectiveness to conventional face-to-face programmes, particularly regarding pain reduction and disability improvement [36,37,38]. Notably, no adverse outcomes have been reported in comparison with face-to-face treatment [36,37,38].

This study aims to analyse the cost-effectiveness and cost-utility of a self-administered telerehabilitation programme based on McKenzie cervical exercises and electroanalgesia compared with the same programme delivered face-to-face in patients with NSCNP. The main objectives are to (1) determine the average cost per patient associated with each intervention; and (2) calculate and report the incremental costs and benefits associated with the implementation of the intervention via telerehabilitation in comparison with the face-to-face intervention.

## 2. Materials and Methods

### 2.1. Study Design and Ethical Approval

This study protocol will be an economic evaluation, comprising a cost-effectiveness (CEA) and cost-utility analysis (CUA), conducted alongside a multicentre, double-blind, randomised clinical trial with two intervention arms. This study will analyse the cost-effectiveness and cost-utility of a telerehabilitation programme (McKenzie exercises combined with electroanalgesia) in patients aged 30–65 years old with NSCNP compared with a control group receiving the same face-to-face programme. Participants will be randomly assigned to one of the two groups (telerehabilitation or face-to-face) in a 1:1 ratio. This protocol has been developed in accordance with the recommendations for interventional trials guidelines (SPIRIT). This study will be conducted in collaboration with the Andalusian Health Service and the Department of Nursing, Physiotherapy, and Medicine at the University of Almeria. Ethical approval was obtained from the Andalusian Biomedical Research Ethics Coordinating Committee (SICEIA-2024-000820-F3) in September 2024.

### 2.2. Recruitment and Inclusion Criteria

A total of 210 participants (*N* = 210) with NSCNP will be recruited from the physiotherapy units of the Torrecardenas-Almeria Hospital Complex, the Granada University Hospital Complex, and the Malaga Health District. Men and women aged between 30 and 65 years with a medical diagnosis of NSCNP, who will agree to participate voluntarily by signing an informed consent form, and who are not receiving physiotherapy treatment at the time of the study will be eligible for inclusion. Patients undergoing rehabilitation treatment for cervical pathologies, those with cervical osteosynthesis material, as well as individuals with cardiac complications, epilepsy, or neoplastic conditions, or who have received radiotherapy in the past six months, will be excluded. All eligible patients will confirm their participation by signing the informed consent form prior to an explanation of the study objectives and methodology being provided by a trained assessor who will be blinded to the treatment allocations.

### 2.3. Randomisation and Blinding

The selected sample (*N* = 210) will be randomly divided into two groups: one group will receive treatment through a telerehabilitation programme based on the exercise protocols of the McKenzie method combined with analgesic electrotherapy, while the control group will follow the same face-to-face programme. This allocation will be performed in an equitable and blinded manner using a list of random numbers generated by Epidat 4.2 software. These cards with individual numbers will be folded and placed in opaque envelopes so that a blinded member of the study can subsequently open the envelope and assign the subject to one of the intervention groups. Each group (telerehabilitation and control) will be divided into three subgroups according to the McKenzie classification: postural, dysfunction, and derangement. The evaluator and the statistician of the study will remain blinded throughout the entire study. The assessor will not attempt to identify the participants’ group assignments, and the data provided to the statistician will be anonymised to ensure confidentiality and unbiased analysis. To ensure anonymisation, any identifiable personal information will be deleted, and a unique ID will be assigned by the principal investigator to each participant before the data are sent to the statistician. The file containing the non-encrypted information will be kept by the principal researcher. Due to the nature of the interventions, neither the participants nor the principal researcher responsible for the treatment will be blinded.

### 2.4. Interventions

After the initial assessment, all participants will receive 3 sessions per week, for a total of 24 sessions. Patients will be instructed in the use of a portable TENS device with a specific analgesic programme for cervical pain (ENRAF NONIUS Iberica, S.A., Madrid, Spain) during the performance of the McKenzie exercises. The specific programme will consist of a conventional low-frequency, long-phase duration TENS (80 Hz/200 μs) applied directly to the cervical area using four electrodes (5 × 5 cm) positioned bilaterally at the paravertebral level. In patients who present with radicular pain, the electrodes will be placed along the pathway of the affected nerve. First, the threshold for detecting the electrical stimulus will be evaluated by increasing the amplitude until the patient indicates sensory perception of the stimulus. Subsequently, the patient will be instructed to increase the intensity of the electrical stimulus to reduce pain during the exercise in accordance with their subjective perception. The electrostimulation treatment will have a duration equal to the total McKenzie exercise protocol. Participants in the telerehabilitation group will be provided with the necessary equipment (tablets, TENS devices, electrodes, and batteries) for the remote delivery of therapy.

The McKenzie exercises that will be performed simultaneously with the electrical stimulation are designed to effect changes in the internal periarticular components of the spinal column. The exercise programme will be as described by Robin McKenzie [39]: 1. Cervical retraction in sitting position; 2. Cervical extension in sitting position; 3. Cervical retraction in supine position; 4. Cervical extension in supine position; 5. Cervical lateral flexion; 6. Cervical rotation in sitting position; 7. Cervical flexion in sitting position.

The telerehabilitation programme will use a support system for the treatment of cervical pain based on web technologies, accredited as a healthcare website. This system has a structure based on four sections: database management, database user profiles, recommendations, and feedback/biofeedback procedures. This system allows for registering and entering a subject and modifying a treatment with electroanalgesia and exercises, according to the symptomatic evolution of the pain. It is based on an initial patient assessment system. Once the initial diagnosis of the patient is made according to the McKenzie clinical subtypes of NP, a multimedia database will be developed with examples of specific treatments (according to symptom evolution) for postural syndrome, dysfunction syndrome, and derangement syndrome. Patients will use their computer or mobile device connected to the internet to access the platform, where they will find the videos of the combined application of electroanalgesia and exercises. The treatments will be provided by the system. The database is configured to accommodate the application of electroanalgesia and exercises based on the diagnosis according to the McKenzie method. Each subgroup corresponds to a programme and a set of previously defined exercises with electroanalgesia as well as recommendations for daily activities, such as manual activity, sitting, standing, or ergonomic postures for daily activities. Therefore, each treatment will use a feature vector VT with 3 characteristics (one for each subgroup), where each position will contain a value indicating the degree of difficulty for each treatment subgroup. The system calculates and recalculates recommendations and updates the values of the relevance estimates for the recommended treatments. The system will recommend treatment Y for subject X, and depending on the modification or remission of the pain, at the start of each session, it will continue with the same plan or will be adjusted following the therapeutic algorithm of the McKenzie method. Once the treatment is completed, the outcome measures will be evaluated. This evaluation will be recorded in the system to be subsequently available in the recommendation processes. Furthermore, the system recalculates the evaluations provided by the patients. To facilitate the correct execution of the treatment, each element on the website is arranged in the order in which it should be performed and represented by icons that are easily associated with each step. This ensures that patients are guided intuitively as they scroll through the website. To verify attendance and ensure that patients complete all sessions properly, patients must enter their ID in the field provided on the website at the end of each session. This confirmation will be emailed automatically to the responsible clinician.

Patients in the face-to-face programme will receive the same treatment as described above, delivered face-to-face by six therapists, each with over 10 years of experience in both intervention procedures. This programme will be developed at the rehabilitation and physiotherapy services of the Andalusian Health Service (Almería, Granada and Málaga), Spain.

### 2.5. Data Collection

Measurements will be taken at three timepoints: before the start of treatment (baseline), after completing the eight-week intervention (post-treatment), and six months after the final session (follow-up). Patients from both groups will be scheduled to attend in person to complete the questionnaires at each assessment; demographic and clinical data, healthcare resource utilisation, associated costs, and sick leave will be recorded.

### 2.6. Outcomes

The main outcome measure will be the healthcare resources used during the study, and their costs will be collected through questionnaires completed by the participants at baseline, post-treatment, and follow-up (6 months). Table 1 provides a detailed list of the relevant resource categories, the sources from which usage data are obtained, and the associated unit costs, Table 2 shows timepoints of assessments. The cost analysis will include resources and expenses related to primary and hospital care, diagnostic testing, and other direct out-of-pocket expenses incurred by the participants (e.g., transportation costs). In addition, the social costs associated with absenteeism and loss of productivity will be included as secondary outcomes. For each participant, information on their resource utilisation will be tracked by means of a receipt log. At each assessment, patients will be asked to recall and detail their use of the described resources during the preceding months. These data will then be converted into costs based on updated unit prices from official sources.

### 2.7. Sample Size and Data Analysis

The sample is calculated with the sample size calculation of Willan [40]. Like many of the parameters that were used, these were unknown, and assumptions were made about health parameters as the difference between the average cost of both treatments was assumed in monetary terms from EUR 300, and the standard deviation of the monetary costs was EUR 600. In addition, the effect of the intervention and the difference between means, to be detected in NDI, is 7 units, having a common variance in NDI in patients with neck pain of 10, which is a clear overestimation of the standard deviation. The correlation coefficient between effectiveness and cost is 0.1, clearly underestimated so that the sample size is increased, with an alpha error of 0.05 and a power of 80%. With these conditions, the sample size was 105 patients with neck pain per group (telerehabilitation programme group and face-to-face programme group). In each group, these patients will be divided into three subgroups (postural, dysfunction, and derangements syndromes).

We will register the number of days that patients are on sick leave due to neck pain, family cost, primary and secondary healthcare consultations (Andalusian Health Service provider), diagnostic test (Andalusian Health Service provider), additional medical services (Andalusian Health Service provider), material provided in intervention (production cost), and direct non-healthcare cost (aid to patients who face disabilities), and work absenteeism cost will be measured using days of work sick leave by salary.

### 2.8. Economic Evaluation

An economic analysis will be performed in STATA 19, including a CEA and a CUA, with the aim of estimating and comparing the incremental costs and benefits of the telerehabilitation and face-to-face care programmes. A six-month time horizon was chosen to capture not only the immediate effects of the interventions but also the costs and outcomes that arise during the months following their implementation. This duration allows a more comprehensive assessment of the interventions’ impact from a societal perspective. Missing cost data will be handled using appropriate techniques, such as multiple imputation, according to the type and magnitude of the missing data. We will use multiple imputation by chained equations. The imputation model will include all variables used in the CEA and CUA, assuming that data are missing at random. We will generate 20 imputed datasets, each analysed separately using the same statistical procedures. The results will then be pooled according to Rubin’s rules, providing unbiased estimates of variances that account for the uncertainty associated with imputed values. Given that the cost distribution is usually skewed due to the presence of patients with very high costs, the mean cost per patient will be presented together with confidence intervals obtained using non-parametric bootstrap methods [41]. QALYs will be calculated as the area under the curve connecting the utility scores recorded at baseline and at subsequent follow-up points. The results will be expressed as incremental cost-effectiveness ratios (ICERs), which reflect the additional cost per each additional unit of outcome achieved. An incremental analysis will be performed to calculate the difference in costs and the difference in outcomes (improvements in effectiveness measured in QALYs) associated with the telerehabilitation and face-to-face programmes. In order to address the uncertainty associated with sample variability, the joint distribution of the differences in costs and effects will be estimated through multiple simulations using non-parametric bootstrap methods. The simulated cost-effectiveness pairs will be graphically represented on a cost-effectiveness plane, and acceptability curves will be generated to reflect the probability that each alternative is cost-effective according to different willingness-to-pay thresholds per QALY [42]. In Spain, the value of a QALY is estimated to be between EUR 22,000 and EUR 25,000 [43]. A sensitivity analysis will be carried out to assess the robustness of the results against different values, assumptions, and methodological approaches [44]. As the time frame of this study is limited, it will not be necessary to apply discounting to costs or benefits. The economic analysis will be reported in accordance with the guidelines of the Consolidated Health Economic Evaluation Reporting Standards (CHEERS) [45].

### 2.9. Adverse Effects

Both treatments are considered safe and, in most cases, well tolerated by participants. Adverse events are defined as unwanted and involuntary signs, symptoms, or conditions that may occur following the intervention, regardless of whether there is a direct causal relationship with the treatment. To date, no significant risks associated with the intervention have been reported. Electroanalgesia is regarded as a low-risk technique. Although no significant adverse reactions have been reported, mild effects such as skin irritation or transient redness at the site of electrode placement or mild allergic reactions to the materials used may occur, which usually resolve without medical intervention. Regarding the exercises, these will be prescribed on an individualised basis following the patient’s assessment, thereby minimising the risk of side effects. Nevertheless, improper execution could cause transient muscular discomfort, which is generally mild and resolves spontaneously within 48 h. Mild muscle fatigue may also occur, which subsides with adequate rest.

All potential adverse effects will be recorded by the investigators. If they occur, the frequency of occurrence will be analysed between the intervention groups. Participants will also be informed of these possible effects during the informed consent process. They will have the option of contacting the research team, either by telephone or email, should they have any questions or experience any symptoms not related to the expected progression. Furthermore, regular reviews of safety protocols will be conducted to ensure the well-being and protection of all participants. In the event of severe adverse effects, the participant will be withdrawn from the study and referred to the pertinent medical unit. Due to the very low risk of severe adverse effects, the approval of the ethics committee, and the close supervision of the research team, the involvement of an external data monitoring committee was considered unnecessary.

### 2.10. Limitations

One of the main limitations of this study is the difficulty in comparing our future results with other similar studies, as evidence in this field is still scarce. Furthermore, the assessment on which we will base our long-term results will be taken 6 months after the end of the intervention. Although we believe that this is adequate, an assessment at 12 months could yield more representative results.

## 3. Ethics and Dissemination

All participants will receive detailed information, both verbal and written, about the study before giving their consent to participate. They will be told that they have the freedom to leave the study at any time without consequences. Those who agree to participate must sign two copies of the informed consent form: one will be retained by the research and evaluation team, and the other will be for the participant’s personal records. All physical documents will remain under strict confidentiality in a locked filing cabinet in the research group’s office, while electronic data will be stored in a password-protected database. The research team will ensure the confidentiality and integrity of all information collected in the study.

Participant data, group allocations, treatment records, and sociodemographic information will be encoded. In addition, the results of the questionnaires will be assessed and scored. The assessor will be responsible for securely storing the collected information, using a locked cabinet for physical documents and a password-protected computer for digital files, restricting access solely to the designated assessor.

Once the first participant has been registered, the inclusion criteria, outcome measures, and analyses will not be modified. Any changes to the protocol, including possible adjustments to the inclusion criteria, outcomes, or analyses, will be communicated to the pertinent Research Committee and will be notified in publications and presentations.

The results of the study, including those related to feasibility, will be published in a peer-reviewed scientific journal. The presentation of the results will be conducted in group format, and the confidentiality of individual data will be preserved in all public communications.

### Trial Status

The protocol was approved on June 2025 by Information System of the Ethics Committees of Research in Andalusia-Spain (SICEIA-2024-000820-F3). The initial recruitment date will be 1 July 2025, and the approximate date of completion of the study will be 30 July 2026.

## 4. Discussion

Therapeutic exercise has become established as a fundamental pillar in the treatment of CNP, with well-designed exercise programmes having been shown to significantly reduce pain, improve cervical function, and decrease associated disability [46,47]. Furthermore, strengthening, stabilisation, and mobility exercises—especially those targeting the deep cervical muscles—have demonstrated sustained positive effects, not only in pain relief but also in improving motor control and quality of life [48,49]. However, although TENS is a therapeutic option used in the management of CNP, current evidence does not support firm conclusions to be drawn regarding its effectiveness. Available studies reveal significant methodological shortcomings and inconsistent results, highlighting the need for high-quality studies [50,51].

Several studies have reported a high level of social acceptance and confidence among patients in the use of telemedicine, especially in contexts of real-time care, remote diagnosis, and treatment [52]. In the musculoskeletal field, telemedicine programmes represent a promising alternative to face-to-face physiotherapy, achieving comparable levels of satisfaction and adherence and also demonstrating a reduction in healthcare costs across various types of conditions [53]. Although telerehabilitation has shown some good results and may be a feasible alternative to face-to-face interventions in different pathologies, its applicability is not fully established yet. This trial aims to contribute high quality evidence to address this gap. One of the main challenges identified in the literature is the absence of CEAs regarding NSCNP and the lack of long-term evaluations; therefore [54,55,56], the final data collection for this study will be extended to 6 months post-intervention.

## 5. Conclusions

Physiotherapy delivered via telerehabilitation is an innovative option for managing NSCNP, with growing interest in its clinical and economic efficiency compared to traditional face-to-face approaches. This study proposes the first randomised clinical trial to evaluate the efficacy, cost-effectiveness, and cost-utility of a combined cervical exercise and analgesic electrotherapy programme delivered simultaneously via telerehabilitation in patients with NSCNP. In addition, the trial has the potential to generate relevant data on the short- and medium-term economic efficiency of telerehabilitation, which will facilitate the assessment of the viability of digital tools in rehabilitation processes. Improving symptoms without interrupting work activity and eliminating geographical barriers may contribute to the sustainability of the healthcare system by shortening waiting lists, reducing costs for patients and companies, and optimising access to treatment.

## Figures and Tables

**Table 1:** Cost measures.

Costs	Breakdown	Source of Cost/Unit	Cost Calculation
Direct Medical Costs			
Primary care	General medicine	Andalusian Health Service	No. of visits × rate per visit
	Nursing		
	Physiotherapy		
Emergency hospital visits	General medicine	Andalusian Health Service	No. of visits × value per visit
	Nursing		
Referrals to services	Traumatology, Rehabilitation, etc.	Andalusian Health Service	Specialty × fee per visit
Home assistance	Ambulance	Andalusian Health Service	Service × fee-for-service
	General medicine		
	Nursing		
Diagnostic tests	X-rays, MRI, CAT Scans, etc.	Andalusian Health Service	Test × test price
Medicines	Muscle relaxants, analgesics, NSAIDs, etc.	Standard drug prices according to the Official General Council of Pharmacists	Medicine × price of the medicine
Non-medical direct costs			
Patient’s transport expenses	Bus	Patient	Number of times × price each time
	Taxi		
	Own transport (petrol)		
Indirect costs			
Absenteeism	Sick leave days	Patient and according to the assigned professional classification (National Statistics Institute of Spain)	Days of sick leave × daily wage

Magnetic resonance imaging (MRI), computed axial tomography (CAT), non-steroidal anti-inflammatory drugs (NSAIDs).

**Table 2:** Timepoints of assessments.

Timepoint	Study Moment										
	Enrolment	Treatment								Follow-up	
	0W	1W	2W	3W	4W	5W	6W	7W	8W	2M	6M
	July 2025	July–September 2025								September 2025	January 2026
Screening and enrolment											
Eligibility screening	X										
Informed consent	X										
Allocation	X										
McKenzie classification	X										
Interventions											
Telerehabilitation programme (experimental)		3 Times per week									
Face-to-face programme (control)		3 Times per week									
Assessments											
Demographic variables		X									
Economic evaluation		X								X	X

## Data Availability

No new data have been created or analysed yet in this study. Data sharing is not applicable to this article.

## Abbreviations

The following abbreviations are used in this manuscript:

NP	Neck pain.
CNP	Chronic neck pain
NSCNP	Non-specific neck pain
TENS	Transcutaneous electrical nerve stimulation
SPIRIT	Standard protocol items: recommendations for interventional trials
NDI	Neck disability index
CEA	Cost-effectiveness analysis
CUA	Cost-utility analysis
QALY	Quality adjusted life years
ICER	Incremental cost-effectiveness ratio
CHEERS	Consolidated health economic evaluation reporting standards
